# Billing Implications of Emergency Medicine Resident Physicians’ Laceration Length Estimates

**DOI:** 10.51894/001c.5065

**Published:** 2016-10-24

**Authors:** Joseph Sherer, Roya Caloia, William D. Corser, Kevin Tishkowski

**Affiliations:** 1 ProMedica Monroe Regional Hospital, Emergency Medicine Residency Program; 2 Michigan State University College of Osteopathic Medicine, Statewide Campus System

**Keywords:** emergency medicine, emergency department billing, laceration estimates

## Abstract

**CONTEXT:**

Lacerations are a common occurrence in urgent care and emergency room settings. The types of lacerations repaired in these settings range from superficial and linear to deep and stellate. Healthcare professionals are required to describe these wounds in documentation and part of that description is length. In a busy clinical setting, many providers use a visual estimation of wound length for documentation. The purpose of this exploratory pilot study was to systematically examine the factors (e.g., sex, residency year, prior laceration training) associated with overall accuracy of five laceration length estimates made on a series of five identically-marked linear dummy torso sutured lacerations by a convenience sample of Emergency Medicine (EM) resident physicians. Before the study, the authors hypothesized that laceration estimates from later-year residents and/or those with more prior laceration training would be more accurate.

**METHODS:**

The EM residents who attended a statewide educational session were encouraged to participate in the study by independently entering information concerning their a) personal characteristics, and b) five laceration length estimates from five dummy torso sutured lacerations onto hard copy forms during break and lunch periods of the daylong conference. The use of any types of measurement devices was prohibited.

**RESULTS:**

A total non-probability convenience sample of 107 participants (93 EM resident physicians and 14 medical student attendees) from 14 different Michigan-based EM residency programs completed a 10-item survey during the educational conference. Results for both composite and individual actual-to-estimated (AE) laceration differences varied widely within the sample, with up to 58.9% of laceration over estimates hypothetically having resulted in overbilling of payers for the laceration repair.

**CONCLUSIONS:**

The considerable range in laceration estimates obtained from these EM clinicians indicate the complexity of attempting to estimate lacerations without measuring devices, as well as the potential for over-billing under such conditions. Larger resident samples recording laceration length estimates, with testing of potential interaction effects on AE patterns, are needed in the future to provide additional evidence concerning this aspect of EM billing.

## INTRODUCTION

Lacerations that require surgical repair are a common occurrence in urgent care and emergency room settings.^1^ The types of lacerations repaired in such settings can range from superficial and linear to deep and stellate wounds. Healthcare professionals such as physicians and nurses are routinely required to describe these wounds in medical record documentation in terms of their length.^1,2^ In busy Emergency Medicine (EM) clinical settings, however, many providers perform a visual estimation of wound length for surgical wound management and insurer documentation. Information concerning EM wound assessments also may be pertinent when phone referrals are made to other specialist providers often expected to evaluate the likelihood of associated nerve, vessel, and ligament or tendon involvement.^3^

Several studies to date have demonstrated the complexity of this clinical phenomenon in EM settings. For example, Peterson, Stevenson and Sahni investigated the predictive influences associated with laceration estimates of fifty EM physicians using a series of seven photographic wound images.^3^ Each participant in this study was asked to enter their respective size estimate from the images provided. In this particular study, a high incidence of estimate error and inter-observer variability was noted between males and females. Overall, male physicians were more likely to overestimate the size and length of wounds, while female providers tended to underestimate characteristics of the same set of wound images.

In 2012, a fairly similar project was conducted by Umbrello et al. with 190 patients in a pediatric emergency department.^4^ Each laceration was visually evaluated by both a resident physician or mid-level provider (i.e., nurse practitioner or physician assistant) as well as an attending EM physician before formal measurements with a measuring device were made. In this study, there were no statistically significant differences in laceration length estimates demonstrated between attending physicians and other types of providers (p=0.583). Notably, however, over 8% of lacerations were misclassified in terms of their lengths, with 20% of facial lacerations billed incorrectly and *upcoded*. Lacerations that had occurred elsewhere on children’s bodies were downcoded in approximately 27% of cases.^4^

Another smaller study in 2014 enrolled 32 EM physicians (nine attending and 23 residents) and 16 nurses. All providers in this sample tended to overestimate the actual length of seven lacerations on pig’s ears and feet.^1^ Notably, both physicians and nurses were more likely to make estimates within the accuracy parameters (i.e., greater than or less than 0.5 cm off) observed by the study team for shorter length lacerations regardless of provider sex or physician-nurse status.^1^

The complex process of learning these types of medical skills begins in medical school, ideally becoming more fully developed during residency. Misestimating lacerations has billing implications and failing to demonstrate improvement during residency could reflect residency training weaknesses.

## STUDY PURPOSE

The purpose of this exploratory pilot study was to systematically examine the factors associated with overall actual-to-estimated (AE) differences of five identically-marked dummy torso sutured lacerations from a convenience sample of EM resident physicians. Respondents from different EM residency programs affiliated with the Michigan State University Statewide Campus System ^5^ confidentially provided their laceration estimates during a regularly scheduled daylong statewide resident meeting. Before the study, the authors had hypothesized that laceration estimates from later-year residents and/or those with more prior training would be more accurate.

In addition to self-recorded linear laceration estimates, residents were asked about: a) current year of residency, b) sex, c) whether or not they had received any prior laceration length training, d) the estimated number of years since last laceration length training (if applicable), e) approximate number of lacerations they saw each month, and e) the name of their EM residency program.

## METHOD

The study obtained campus-based institutional review board approval before any data collection was begun. A total of between 200 and 300 EM residents generally attend monthly statewide resident meetings, and data collection was scheduled for the November 2015 educational session. Those EM residents who attended this session were encouraged by the first author to participate in the study by independently and directly entering information on a hard copy survey form.

Information concerning participants’ personal characteristics, and their laceration length estimates from five identical dummy torsos was collected during break and lunch periods. Interested residents were directed by the first author to three study tables that had been set up outside of the main conference room. No measuring devices of any type were allowed during respondents’ data entry as they confidentially entered their study data.

The first two authors (JS and RC) had precisely measured the actual length of each dummy torso laceration before the educational session. The six dummy torsos were marked with absolute linear lacerations of the following sizes: a) 3.0 cm, b) 8.0 cm, c) 13.0 cm, d) 21.0 cm, and e) 31.0 cm.

The final project data set was entered by the first author into a password protected data set. The third author then securely received and stored an electronic copy of the raw data set and created a cleaned working study data set for analyses. Calculations were completed for each respondent’s AE laceration differences for each of their five laceration measurements.

After data set cleaning, a series of descriptive statistical analyses were completed before doing inferential analyses, to identify possible subgroup AE measurement error differences by the selected resident characteristics, observing a p-value significance level of 0.05. AE difference data were used to calculate the proportion of times in which residents had misestimated lacerations that could hypothetically result in under-billing or overbilling while observing *Current Therapeutic Technology* (CPT) parameters.^6^

The CPT code set is maintained by the American Medical Association through the CPT Editorial Panel. The CPT code set describes medical, surgical, and diagnostic services and is designed to communicate uniform information about medical services and procedures among physicians, coders, patients, accreditation organizations, and payers for administrative, financial, and analytical purposes. CPT codes are similar to *International Classification of Disease Coding* (ICD-9 and ICD-10) coding,^7^ although they specify the services rendered, rather than the patients’ diagnosis. Laceration repairs in the Emergency Department are billed according to CPT Codes.

Lacerations are billed a *standard amount*, which is then adjusted using a *geographical* c*orrector*, meaning that the actual amount billed will vary from region to region within the United States. Lacerations are billed based on length and complexity of repair (i.e., simple, intermediate, complex). The lacerations are billed according to the following length increments: 0-2.5 cm, 2.6-7.5 cm, 7.6-12.5 cm, 12.6-20 cm, 20.1-30 cm, and > 30 cm.

A *simple laceration* is defined in CPT Codes (12001 – 12021) as a wound that is superficial without significant involvement of deeper structures and requiring a simple one layer closure.

An *intermediate repair* (codes 12031 – 12057) includes layered closure of one or more of the deeper layers of subcutaneous tissue and superficial (non-muscle) fascia in addition to the skin (epidermal and dermal) closure. Single-layer closure of heavily contaminated wounds that require extensive cleaning or removal of particulate matter also constitute intermediate repairs.

A *complex repair* (codes 13100 – 13160) includes the repair of wounds requiring more than layered closure, such as traumatic lacerations or avulsions, extensive undermining, stents, or retention sutures. A complex repair code is the most complicated surgical repair that a physician will perform on the integumentary system. The physician would have to perform more than layered closure in order to bill for such a complex repair.

In order to correctly code and bill a laceration repair, the documented length of the laceration must be accurately measured, along with the complexity of the repair.

## RESULTS

### Descriptive Statistics

All study analyses were conducted using SPSS version 22 analytic software.^8^ A total non-probability convenience sample of 107 EM resident physicians (this total including 14 medical student attendees) completed the 10-item survey questionnaire that had been created by the first two authors for this study. Seventy-two (67.3%) of total respondents were male and 28 (26.2%) respondents reported having received some form of past laceration estimate training. Those respondents who reported having received prior training estimated they had received such training an average of about two and a half years earlier, although this ranged from zero to eight years before the time of survey. Responding residents were enrolled in 14 different Michigan-based EM residency programs, and estimated they had seen a monthly average of 7.96 (SD 5.04) lacerations in clinical practice, although this number did range considerably from two to 30. Other respondent characteristics are listed in Table 1.

**Table 1.** Respondent Characteristics (N = 107 Emergency Medicine residents

Characteristic	n	Percent
**Sex**		
Male	72	67.3
Female	35	32.7
**Has Respondent Ever Received Any Prior laceration training?**		
Yes	79	73.8
No	28	26.2
**Year of residency**		
Medical student	14	13.1
PGY-1	34	31.8
PGY-2	23	21.5
PGY-3	8	7.5
PGY-4 or PGY-5	19	17.8
Missing	9	8.4
**Number of lacerations seen per month**		
Two to five	44	41.1
Six to nine	21	19.6
Ten or more	41	38.3
Missing	1	0.9

**Table 2.** Sample Laceration Estimate Differences of (n = 107 Emergency Medicine residents)

Raw Laceration Estimates	
Laceration ONE (actual length 8.0 cm.)	Mean 8.46 cm
	(SD 2.50)
	Range 2.54 to 17.78
Laceration TWO (actual length 3.0 cm.)	Mean 3.43 cm
	(SD 0.97)
	Range 2.00 to 9.00
Laceration THREE (actual length 13.0 cm.)	Mean 13.85 cm
	(SD 4.03)
	Range 3.00 to 25.40
Laceration FOUR (actual length 21.0 cm.)	Mean 22.75 cm
	(SD 6.17)
	Range 11.00 to 40.64
Laceration FIVE (actual length 31.0 cm.)	Mean 32.87 cm
	(SD 9.50)
	Range 12.00 to 60.96
Total Net AE * Laceration Estimate Difference	Mean 4.86 cm
	(SD 21.59)
	Range -61.00 to 68.70
Laceration ONE Difference (actual length 8.0 cm.)	Mean 0.49 cm
	(SD 2.50)
	Range -5.46 to 9.78
Laceration TWO Difference (actual length 3.0 cm.)	Mean 0.46 cm
	(SD 0.95)
	Range -1.00 to 6.00
Laceration THREE Difference (actual length 13.0 cm.)	Mean 0.79 cm
	(SD 4.23)
	Range -10.00 to 12.40
Laceration FOUR Difference (actual length 21.0 cm.)	Mean 1.75 cm
	(SD 6.17)
	Range -10.00 to 19.64
Laceration FIVE Difference (actual length 31.0 cm.)	Mean 1.51 cm
	(SD 10.16)
	Range -20.00 to 29.96

* AE = Actual-to-Estimated Difference

### Data Cleaning and Analyses

After data cleaning was completed with over 95% complete data, a series of descriptive statistics were generated. The cleaned data were examined for distributional patterns of AE laceration length differences. These data then were used to determine whether any laceration estimates may have resulted in laceration repair under-billing or over-billing coding. A series of initial cross-tabulation charts with analysis of variance (ANOVA) procedures ^9^ were generated to examine for potential significant sample subgroup (e.g., males vs. females, less or more-experienced) AE differences and correlations.

The overall distribution of individual laceration differences and composite differences (i.e., combined total of AE differences from each respondent) appeared to be quite non-parametric (not normally distributed) using analytic software graphics. The characteristics of six specific outlier respondents (i.e., those with a notably wider range of AE estimate totals) were compared to the rest of the sample, but retained in the final analytic sample since these respondents’ characteristics were similar to the rest of the sample.

Individual AE differences were entered into new data set fields for analyses, including comparisons of: a) year of residency (categorical variable), b) sex (categorical), c) whether or not respondent had received any prior laceration training (dichotomous), and d) categorized monthly estimated numbers of lacerations respondent had seen during recent months. (The distribution of participants across the 14 different reported residency programs was clearly too broad for the authors to include this possible term into analytic models). To capitalize on the broader distribution of the laceration estimates, AE data were included in primary analytic models as both continuous and categorical variable study outcomes.

Finally, a series of six multinomial logistic regression modeling (MLM) procedures were run (one model for total combined AE differences and one model for each of the five individual laceration AE differences) to examine the potential predictive significance of the four selected key categorical factors on AE differences.^10^ These types of analytic procedures are especially appropriate for smaller samples in which outcome cell frequencies are lower and not normally distributed.^10^

After conducting several forms of MLM models, it was concluded that none of the selected terms demonstrated statistical significance (p-values were between 0.063 and 0.917) on individual or composite AE outcomes when treated as categorical variables. Notably, no significant differences in AE laceration differences were found between the 57 first- or second-year residents compared to the 27 third- through fifth-year residents when these data were treated in either continuous or categorical form. It does not appear that any of the earlier cited studies examined this factor for possible significance.

**Figure 1.**
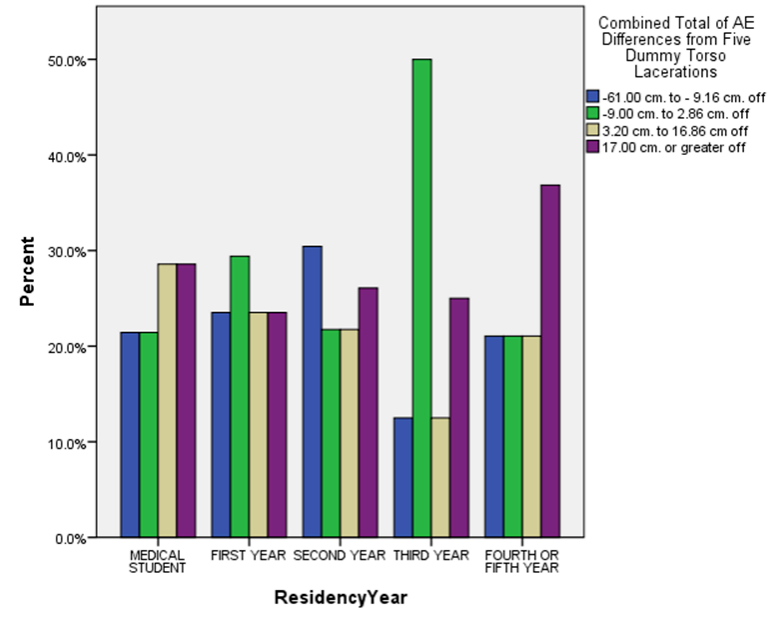
Variations in Total Actual-to-Estimated Laceration Length Differences by Residency Year (n = 107)

Figures 1 through 3 vividly depict some of the most striking study findings when categorized by *total net* percentage AE differences across the five torso lacerations. For example, Figure 1 demonstrates the significant variations in total AE differences after categorizing by respondents’ residency years. A quick review of this figure certainly seems to indicate that AE underestimate differences (blue and green bars) and overestimate differences (tan and purple bars) varied considerably across residency year total AE difference subcategories.

**Figure 2.**
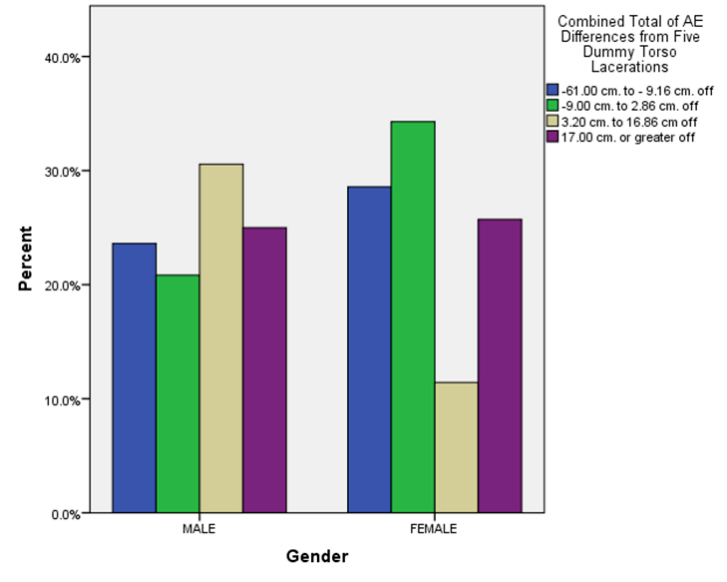
Variations in Total Actual-to-Estimated Laceration Length Differences by Respondent Gender (n = 107)

**Figure 3.**
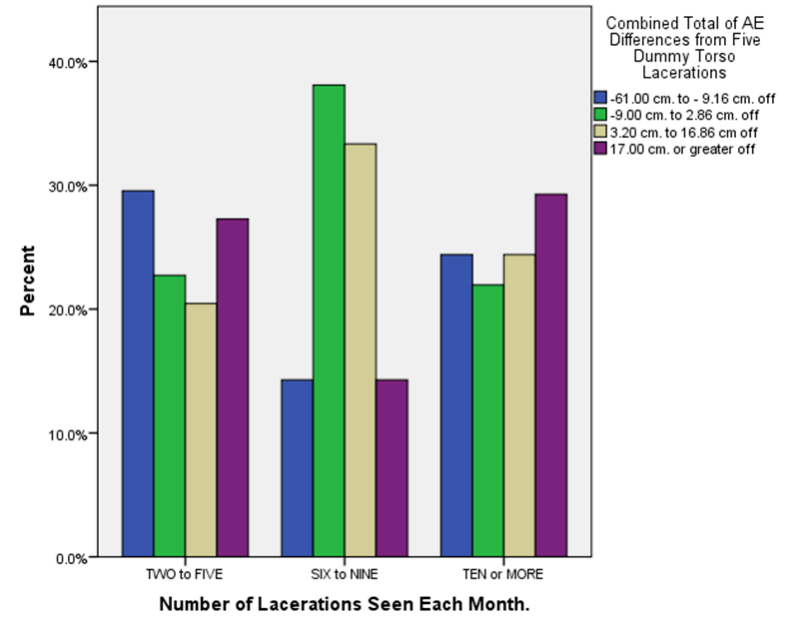
Variations in Total Actual-to-Estimated Laceration Length Differences by Monthly Number of Lacerations seen by Respondent (n = 107)

Similar to one earlier study,^3^ Figure 2 depicts how males (albeit more numerous) disproportionately overestimated laceration lengths compared to female respondents across each AE difference category. A review of Figure 3, however, suggests that seeing more monthly ED lacerations in an ED practice setting may be quite variably associated with less severe under- or over-estimate totals.

In the end, the authors concluded that their inability to detect statistically significant sample subgroup AE differences may have been quite likely due to the lack of statistical power from a sample of this size. These final model results also may have been skewed by other unmeasured factors.

### Prospective Under- or Overbilling

Finally, the authors evaluated what proportion of the respondents’ AE differences could have hypothetically resulted in under-billing or over-billing when observing CPT laceration length parameters. The following proportions of individual laceration estimates likely would have resulted in inaccurate billing claims: a) Laceration One (8.0 cm absolute length) - 49 (45.8% of all estimates), b) Laceration Two (3.0 cm) - 19 (17.8%), c) Laceration Three (13.0 cm) - 56 (52.3%), d) Laceration Four (21.0 cm) - 63 (58.9%), and e) Laceration Five (31.0cm) - 34 (31.8%). Overall, neither the shortest nor longest lacerations were as subject to AE misestimates as those in the middle lengths of lacerations provided to the respondents.

## LIMITATIONS

These initial project results should be reviewed within the context of several clear limitations. These findings are based on data from a smaller self-selected convenience sample of EM residents in a variety of EM residency program settings, although this sample is larger than some of the earlier-cited studies. The project was likely underpowered to detect meaningful AE sample subgroup differences that may have been shown in a larger diverse sample of EM resident respondents.

## CONCLUSIONS

In summary, the considerable range in laceration estimates obtained from this sample of EM resident physicians suggests the complexity of attempting to estimate lacerations without measuring devices. These results also indicate the sizable potential for over-billing of laceration repairs completed in rushed EM clinical care settings.^1^ It was unexpected that some of the factors the authors initially had hypothesized might be more significant in resident physician AE differences each fell out of statistical significance in all of the final predictive models.

Still, the wide AE estimate variations shown in this study suggest that controlled studies with larger samples certainly are warranted. This pilot project did apparently involve the largest sample of providers in the EM literature to date. The findings indicate there is significant potential for overestimation or underestimation of laceration lengths on the part of EM providers that will have sizable billing implications in contemporary health care delivery settings. Future studies with larger resident samples and the testing of potential interaction effects on AE patterns are needed. Ideally, the results of this study will provide initial evidence concerning this routine aspect of EM billing and serve to inform the development of graduate medical training modules and workshops.

### Conflict of Interest

The authors declare no conflict of interest.
